# Supra-threshold auditory brainstem response amplitudes in humans: Test-retest reliability, electrode montage and noise exposure

**DOI:** 10.1016/j.heares.2018.04.002

**Published:** 2018-07

**Authors:** Garreth Prendergast, Wenhe Tu, Hannah Guest, Rebecca E. Millman, Karolina Kluk, Samuel Couth, Kevin J. Munro, Christopher J. Plack

**Affiliations:** a Manchester Centre for Audiology and Deafness, University of Manchester, Manchester Academic Health Science Centre, M13 9PL, UK; b NIHR Manchester Biomedical Research Centre, Central Manchester University Hospitals NHS Foundation Trust, Manchester Academic Health Science Centre, Manchester, M13 9WL, UK; c Department of Psychology, Lancaster University, Lancaster, LA1 4YF, UK

**Keywords:** Auditory brainstem response, Test-retest reliability, Cochlear synaptopathy, Summating potential, Electrode montage

## Abstract

The auditory brainstem response (ABR) is a sub-cortical evoked potential in which a series of well-defined waves occur in the first 10 ms after the onset of an auditory stimulus. Wave V of the ABR, particularly wave V latency, has been shown to be remarkably stable over time in individual listeners. However, little attention has been paid to the reliability of wave I, which reflects auditory nerve activity. This ABR component has attracted interest recently, as wave I amplitude has been identified as a possible non-invasive measure of noise-induced cochlear synaptopathy. The current study aimed to determine whether ABR wave I amplitude has sufficient test-retest reliability to detect impaired auditory nerve function in an otherwise normal-hearing listener. Thirty normal-hearing females were tested, divided equally into low- and high-noise exposure groups. The stimulus was an 80 dB nHL click. ABR recordings were made from the ipsilateral mastoid and from the ear canal (using a tiptrode). Although there was some variability *between* listeners, wave I amplitude had high test-retest reliability, with an intraclass correlation coefficient (ICC) comparable to that for wave V amplitude. There were slight gains in reliability for wave I amplitude when recording from the ear canal (ICC of 0.88) compared to the mastoid (ICC of 0.85). The summating potential (SP) and ratio of SP to wave I were also quantified and found to be much less reliable than measures of wave I and V amplitude. Finally, we found no significant differences in the amplitude of any wave components between low- and high-noise exposure groups. We conclude that, if the other sources of between-subject variability can be controlled, wave I amplitude is sufficiently reliable to accurately characterize individual differences in auditory nerve function.

## Highlights

• ABR wave I and V amplitudes have excellent test-retest reliability in humans.
• SP amplitude and SP/AP ratio have poor test-retest reliability.
• Canal tiptrodes result in only slightly increased reliability re. mastoid electrodes.
• No significant differences in amplitudes between low- and high-noise exposed females.

## 1 Introduction

The auditory brainstem response (ABR) is a well-established diagnostic tool widely used in the clinic to assess auditory function (see Hall, 1992, for an overview). The ABR is evoked by transient stimuli, typically clicks or tone bursts, and consists of a series of waves, with wave I reflecting auditory nerve function, and wave V resulting from generators in the rostral brainstem. The threshold and latency of wave V are the most common clinical metrics of the response. However, wave I has also proved valuable, particularly in research studies, as a more direct measure of peripheral auditory function (Schaette and McAlpine, 2011; Santos et al., 2017).

Wave I amplitude has attracted considerable interest recently, following the demonstration of noise-induced cochlear synaptopathy in the mouse model by Kujawa and Liberman (2009). In the base of the cochlea, up to 50% of synapses between inner hair cells and auditory nerve fibers were destroyed after a 2-h exposure to 100 dB SPL noise (8–16 kHz). Post-exposure measures of absolute auditory sensitivity were unaffected, but histological analyses confirmed the dramatic loss of cochlear synapses. Post-exposure ABR measures showed unaffected responses close to threshold. However, at medium-to-high sound intensities there was a permanent reduction in the amplitude of wave I of the ABR (by 60% at 32 kHz and ∼30% at 12 kHz), reflecting decreased auditory nerve activity.

These results suggest that wave I of the ABR might have potential as a non-invasive measure of cochlear synaptopathy in human listeners. However, the evidence for noise-induced synaptopathy in humans, based on ABR results, is somewhat inconsistent. Recent work from our laboratory has found no evidence that greater lifetime noise exposure, which we assume to be a proxy for greater synaptopathy, is associated with a reduction in ABR amplitude for normal hearing listeners (Prendergast et al., 2017) or listeners with tinnitus (Guest et al., 2017). An absence of a relation between noise exposure and ABR wave I amplitude has also recently been reported by a number of other laboratories using different normal-hearing cohorts (Spankovich et al., 2017; Grinn et al., 2017; Fullbright et al., 2017). Liberman et al. (2016) also reported no significant reduction in wave I amplitude with increasing noise exposure, but did find a significantly increased ratio between the summating potential (SP; reflecting hair cell function) and action potential (AP; equivalent to wave I of the ABR, reflecting auditory nerve function). Bramhall et al. (2017) reported that some groups of firearm users exhibited reduced ABR wave I amplitudes consistent with cochlear synaptopathy and Grose et al. (2017) found a reduced wave I/V ratio in noise-exposed listeners relative to controls. There remain many unanswered questions regarding how these studies can best be reconciled and the extent to which high-frequency hearing loss, gender, and homogeneity of noise exposure can account for the differing evidence for this phenomenon in humans. One additional concern, despite the clear changes in ABR wave I in the animal model of synaptopathy, is whether the ABR is the best tool for identifying these neural changes in the human listener.

If the early waves of the ABR are to have utility as a diagnostic measure in individual listeners, they must be reliable, with low measurement error. As ABR wave I amplitude tends to be lower than wave V amplitude, the response may be more difficult to measure reliably (Mehraei et al., 2016). However, there is little available evidence that addresses this issue directly. Much work on the test-retest reliability of the ABR focuses on the latency of wave V because of its clinical relevance. Edwards et al. (1982) provided an overview of ABR amplitude and latency reliability across a six month period, using 72 dB nHL (72 dB above the normal adult hearing threshold) monaural clicks in 10 listeners. No significant differences emerged between sessions for any wave amplitudes or latencies, or for wave I/V ratios. Using a mean-squared-difference approach, it was found that the participant contributed most variability to the measured responses, followed by ear, session (different days), and run (different acquisition on the same day); however, this was only estimated using wave latency. Lauter and Loomis, 1986, Lauter and Loomis, 1988 tested seven listeners in eight separate weekly sessions and all waves (I-V) were evaluated. The data show high repeatability across the different testing sessions for both amplitude and latency. Rather than a formal assessment of reliability, the approach used the coefficient of variation (CoV; standard deviation divided by the mean) as a marker of “stability” and used ANOVAs to determine that between-subject variability was significantly greater than within-subject variability. Munjal et al. (2016) evaluated the long-term test-retest reliability of the ABR in 50 normal hearing listeners at 3, 6 and 12 month intervals. Only latencies and inter-peak latencies were studied, which demonstrated good reliability overall, although there were differences in the absolute latency of wave I across the different test intervals.

The studies discussed above all used either linear correlations or ANOVAs to estimate the reliability of ABR responses across multiple sessions. These statistical tools are not formal methods of quantifying reliability, unless the ANOVA is set up in an appropriate manner (Zaki et al., 2013; Kim, 2013). A more appropriate method is to use the intra-class correlation coefficient (ICC; Shrout and Fleiss, 1979), which estimates the proportion of the total variance that can be attributed to between-subject variability. Recently, Bidelman et al. (2017) used the ICC to study the test-retest reliability of sub-cortical and cortical auditory evoked potentials. Wave V of the ABR was evaluated, in response to an 80 dB nHL click stimulus, and the amplitude and latency ICCs were 0.65 and 0.76 respectively, reflecting good test-retest reliability.

The primary motivation for the current study was to determine the test-retest reliability of ABR wave I, to evaluate its suitability for measuring auditory nerve function in individual human listeners. There were also a number of secondary questions which the present study was able to address in parallel to the main research question. By using two different EEG montages, a scalp-mounted mastoid electrode and a canal tiptrode (a gold-wrapped foam insert which records the electrical potential from the ear canal), we were able to determine the extent to which reliability is improved by recording from closer to the neural generator of wave I. A canal tiptrode is known to produce a larger wave I response than a scalp-mounted mastoid electrode (Bauch and Olsen, 1990), and it was therefore predicted that the canal tiptrode would produce a more reliable response by virtue of an enhanced signal-to-noise ratio. Furthermore, by using a tiptrode (which emphasizes the SP) we were able to measure the reliability of the SP/AP ratio (utilized by Liberman et al., 2016), and thus evaluate the potential clinical utility of this measure for the detection of synaptopathy.

Finally, the study recruited groups of low- and high-noise exposed female listeners to determine whether changes in the ABR or SP/AP are associated with noise exposure in a single-sex cohort in which audiometric function is tightly controlled. It was predicted that high-noise exposed listeners would yield smaller wave I amplitudes, and larger SP/AP ratios, than low-noise exposed controls.

## 2 Methods

### 2.1 Participants and test sessions

**Fig. 1:**
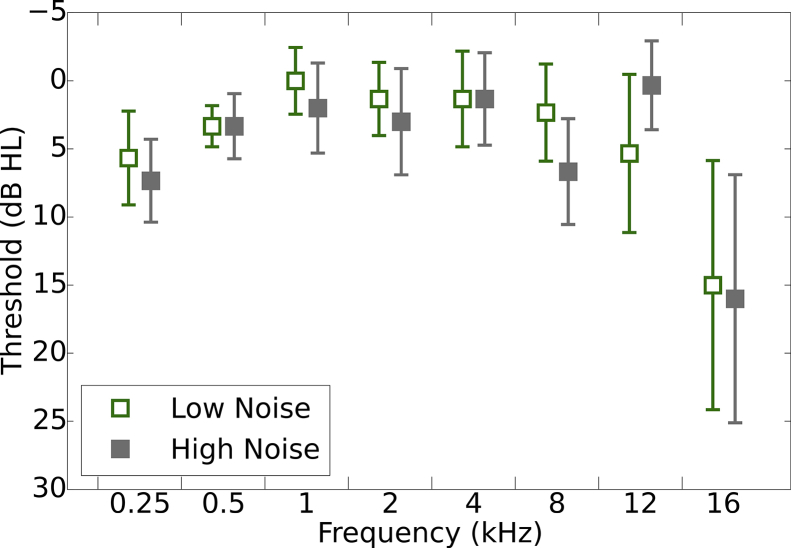
Pure tone air-conduction audiometric thresholds. Thresholds are shown for the test ear, with 95% confidence intervals, for the two groups of listeners. N = 15 in each group.

Thirty female participants were tested, all with clinically normal audiometric thresholds (see section 2.3 and Fig. 1). Participants were recruited into two equal-sized groups based on noise exposure histories (see section 2.2). The mean age of participants in the low-exposure group was 23.87 years (range, 19–31) and in the high-exposure group was 24.87 years (range, 20–34). The study was approved by the University of Manchester Research Ethics Committee (project number 16206) and informed, written consent was obtained from all participants.

Testing was conducted over three sessions. Noise exposure estimates and pure tone audiometry were performed in the first session to establish eligibility. The second session (Test 1, T1) consisted of the ABR and distortion product otoacoustic emission (DPOAE) recordings. The third and final session (Test 2, T2) was a replication of session 2 and was completed on a different day to that of session 2. There were no criteria to constrain how many days elapsed between T1 and T2, provided it was at least 12 h. Each test session took approximately 1 h. The average number of days between test sessions was 3.5 (s.d. = 3.3; range = 1–12) for the low-noise exposure group and 3.3 (s.d. = 2.7; range = 1–8) for the high-noise exposure group.

### 2.2 Noise exposure

Lifetime noise exposure was estimated using a structured interview developed to assess the effectiveness of the UK noise at work regulations (Lutman et al., 2008). The specific implementation used is described fully in Guest et al. (2017). In summary, participants are asked to consider any high-noise (above ∼ 80 dBA) environments/activities to which they have exposed themselves over the course of their lifetime. The duration and frequency of exposure is estimated from discussion with the participant and entered into the following formula:

*U* = 10^(L−*A*−90)/10^ x *Y* x *W* x *D* x *H* / 2080,

where *U* is cumulative noise exposure, *L* is estimated noise exposure level in dBA, *A* is attenuation of hearing protection in dB, *Y* is years of exposure, *W* is weeks of exposure per year, *D* is days of exposure per week, *H* is hours of exposure per day, and 2080 corresponds to the number of hours in a working year. One noise exposure unit is equivalent to exposure for 1 year to a working daily level of 90 dBA. For our purposes, we used the raw units of noise exposure (linearly related to total energy of exposure above 80 dBA) and these were log transformed to produce a normal distribution. Each such logarithmic unit is a factor of 10 in terms of lifetime exposure energy. The cut-off between the low- and high-noise exposure groups was a transformed score of 1.

### 2.3 Pure tone audiometry

Pure tone audiometry was performed in each ear separately at octave frequencies between 0.25 and 8 kHz in accordance with the British Society of Audiology (2011) recommended procedure. Air-conduction thresholds were measured in a sound-attenuating booth using a Kamplex KC50 audiometer coupled to TDH-39P supra-aural headphones. The audiometric criterion for inclusion in the study was audiometric thresholds <25 dB HL in both ears at all standard audiometric frequencies. High-frequency audiometric thresholds were also acquired at 12 and 16 kHz using Sennheiser HDA 300 headphones.

### 2.4 DPOAEs

DPOAEs were acquired from both ears using the Otodynamics ILO v6 clinical OAE software interfaced with a laptop. The ILO probe microphone was calibrated daily using a 1-cc cavity. The frequency ratio of the two primary tones, f_2_/f_1_, was 1.22. Responses were recorded for f_2_ frequencies of 1, 1.5, 2, 3, 4, 6, and 8 kHz. The level of both tones was 70 dB SPL. The cubic distortion product (2f_1_-f_2_) amplitude was used as a measure of the DPOAE. Data collection was terminated after 240 low-noise sweeps had been obtained at each frequency. A signal-to-noise ratio of 3 dB was required for the DPOAE to be identified as present. 4% of the DPOAEs were not present (1.4% from the low-noise exposure group and 2.4% from the high-noise exposure group), and these values were excluded from the average and the calculation of confidence intervals.

### 2.5 ABRs

#### 2.5.1 Recording procedure

Data were recorded using an ICS Chartr EP 200 (Otometrics) and insert earphones supplied with the system. For both montages the positive electrode was placed at Cz. Two different reference electrodes were used; one coupled to the gold-wrapped insert eartip (canal tiptrode) and one standard electrode mounted on the ipsilateral mastoid. An electrode placed on the contralateral mastoid served as the common ground. All electrode impedances were below 5 kΩ and data were sampled at 30 kHz. All recordings were performed by the same researcher to obtain consistent electrode placement, and canal tiptrodes were inserted by the same researcher such that the bottom edge of the foam insert was flush with the start of the ear canal.

Clicks were 100 μs in duration and presented in alternating polarity at 80 dB nHL (115.5 dB peSPL) at a rate of 11/s. Stimuli were presented to the right ear, without the left ear plugged. Signals were amplified with a gain of 50,000 and band-pass filtered between 0.1 and 1.5 kHz (with low- and high-pass roll-offs of 12 dB/octave and 6 dB/octave, respectively). Data were collected over a 20-ms epoch and averaged for a minimum of 6000 repetitions. In sessions 2 and 3 (T1 and T2), two such recordings were made within a 1-h period (with the electrodes remaining attached between recordings). The grand average waveform, taken over both acquisitions, was used to characterize the response on each day. Participants lay in a comfortable position and were asked to remain still during the recordings. Data were acquired in a sound-treated, but not sound-proofed, room.

#### 2.5.2 Response identification

Three waves were identified in each recording: the SP, wave I and wave V. The average waveform for each listener was subjected to an automated peak- and trough-picking procedure based on extracting the phase reversals from the first derivative of the time series (Prendergast et al., 2017). Time windows were constructed around waves I and V and the largest identified peak within the window was selected. The center of the window was determined by the peak in the grand average ABR waveform using all 30 participants and both montages, which were at 1.70 and 5.60 ms for waves I and V, respectively. The edges of the window were set by using standard deviations of ABR latency reported in response to a 70 dB nHL 100 μs click (Issa and Ross, 1995). Standard deviations were 0.17 ms for wave I and 0.21 ms for wave V. The bounds of the windows for our analyses were ±3 standard deviations around the peak central values described above. The SP peak was identified as a peak which occurred 0.5–1.5 ms after stimulus onset. If no peak was present in this time window, it was defined as the point at which the first differential of the waveform within this window was lowest, i.e. when the rate-of-change was closest to a phase reversal. Waves I and V were calculated as peak-to-trough, with the trough constrained to fall within 2/2.5 ms of the identified peak for waves I and V, respectively. If multiple troughs were present, the one which gave the largest peak-to-trough amplitude was used. The SP was defined as being peak-baseline rather than peak-to-trough. The baseline was calculated as the lowest value in the first 1 ms of the waveform (Liberman et al., 2016). To be consistent with Liberman et al. (2016), the AP values used to compute the SP/AP ratio were peak-baseline values rather than the peak-to-trough values more commonly used to characterize wave I. This made little difference to the consistency of the SP/AP ratio across the test sessions. To make this distinction clear the manuscript will use the terms wave I (peak-to-trough) and AP (peak-to-baseline) to differentiate the two measures. These analyses were performed in Python (version 2.7). S1 of Supplementary Materials provides a schematic of how each wave amplitude was calculated.

The peaks were visually inspected to ensure that they appeared to select waves I, V and the SP. It was confirmed that the automated procedure was performing appropriately and it was not necessary to redefine any of the peaks.

### 2.6 Statistical metrics

ANOVAs were used to determine if there were differences in wave amplitudes as a function of noise exposure. The CoV was used as a descriptive statistic of overall variability for the different groups, montages and the waves. For test-retest reliability, Spearman correlation coefficients were used as a descriptive statistic, but the ICC was used to formally quantify the reliability of the measures across the test sessions. The ICC estimates the proportion of total variance that is between-subject rather than between-measurements. The ICC uses pooled scaling and standard deviations for the full dataset rather than for each group independently, and is more robust than Pearson correlation coefficients for estimating the correlation in small sample sizes. Furthermore, the assumptions of linearity implicit in a Pearson correlation coefficients can lead to high correlations in cases where the ICC is in fact poor (McGraw and Wong, 1996). There are a number of different formulations for the ICC. Here ICC1 (as defined by Shrout and Fleiss, 1979) was used when both observations were from the same montage. ICC1 is sensitive to differences in means between the observations and is a measure of absolute agreement. ICC3 was used when comparing observations between montages, which is insensitive to mean differences and the different observations are treated as fixed effects. In all cases, individual responses were treated as single measures rather than considering the reliability of average responses. All statistical analyses were performed in R (R Core Team, 2015).

## 3 Results

### 3.1 Noise exposure

The mean log-transformed noise-exposure score for the low-noise exposure group was −0.98 (std = 1.05; min = −3.00; max = 0.52) and for the high-noise exposure group was 1.55 (std = 0.42; min = 1.08; max = 2.64). The high-noise exposure group had a mean lifetime exposure energy roughly 340 times that of the low-noise exposure group. The difference in exposure between the two groups was due to a combination of both level and duration. The loudest activities reported by the high-noise group were on average 12.5 dB more intense than those of the low-noise group. The high noise group also reported average exposure durations for a single activity which were 2.5 times longer than those of the low-noise group. The high-noise group also typically reported more numerous exposure activities, and so the average total lifetime exposure was three times greater than for the low-noise group. The mean exposure for the low-noise group is equivalent, in terms of total energy, to that for an individual who goes to a nightclub or live music event for 1.5 h, once per year, for five years. The mean high-noise exposure is equivalent to going to the same event for 3 h, three times per week, every week of the year, for five years. These exposure values are comparable to those reported by Guest et al. (2017) and there is clear separation between the groups. The high-noise exposure group for the current study was less exposed than the highest-exposed participants reported by Prendergast et al. (2017). Prendergast et al. (2017) tested a large cohort and inspection of these data indicate that when recruiting largely from a University population, a log exposure value of 1.5 is high for people aged 18–25, with only 12% of people within this group reporting a log exposure score in excess of 1.5.

### 3.2 Pure tone audiometry

Fig. 1 shows pure tone audiometric thresholds for the test ear (right ear) of the two groups. The groups appear to be well matched and a mixed design ANOVA with within-subject factors of Ear (two levels; left, right) and Frequency (eight levels; 0.25, 5, 1, 2, 4, 8, 12, and 16 kHz) and a between-subjects factor of Group (two levels; low- and high-noise exposure) confirmed that there was no main effect of Group (F[1,28] = 1.15; p > 0.05) nor Ear (F[1,28] = 0.22; p > 0.05), but there was a significant main effect of Frequency (F[2,58] = 14.37; p < 0.01). Bonferroni-corrected pairwise comparisons indicated that hearing thresholds at 16 kHz were higher than all other frequencies except 0.25 kHz. Thresholds for 0.25 kHz were higher than those at 0.5, 1, 2 and 4 kHz. A significant three-way interaction between Ear x Frequency x Group was found (F[4122] = 2.68; p < 0.05). The high-noise group showed higher thresholds at 16 kHz compared to the low-noise exposure group in the left ear but not the right ear. Since ABRs were acquired from the right ear, the groups were well matched in terms of audiometric thresholds.

### 3.3 DPOAEs

**Fig. 2:**
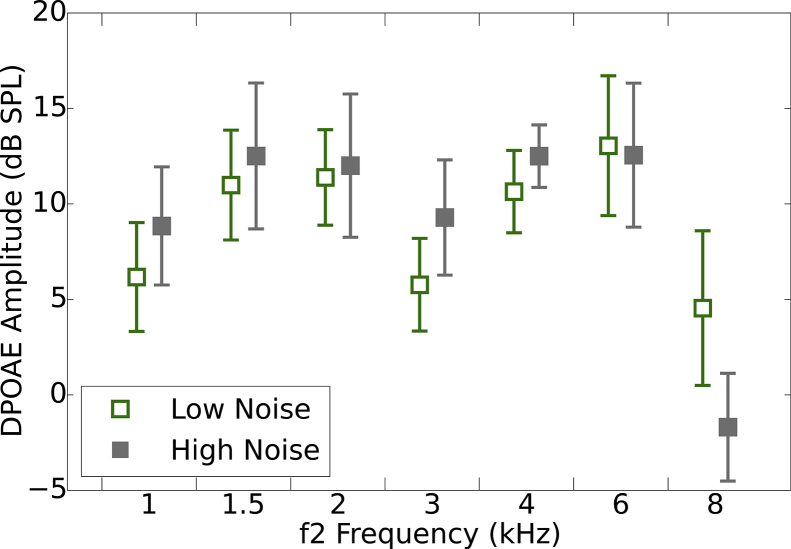
Distortion product otoacoustic emissions. DPOAEs from a single session (T1) for the test ear are shown, with 95% confidence intervals, for the two groups of listeners.

Fig. 2 shows average DPOAE amplitudes for the two groups at each frequency in the test ear (right ear). DPOAEs were collected in the same sessions as the ABR data (T1 and T2). A mixed design ANOVA was used with three within-subject factors of Ear (two levels; left and right), Frequency (seven levels; 1, 1.5, 2, 3, 4, 6, and 8 kHz) and Test-retest (two levels; T1 and T2), and a between-subject factor of Group (two levels; low- and high-noise exposure). There was no significant main effect of Group (F[1,12] = 0.23; p > 0.05), Ear (F[1,12] = 0.24; p > 0.05), nor Test-retest (F[1,12] = 0.18; p > 0.05). A significant main effect of Frequency was found (F[2,28] = 11.12; p < 0.01). Bonferroni-corrected pairwise comparisons indicate that DPOAE amplitudes at 8 kHz were significantly lower than those at 1.5, 2, 4, and 6 kHz. No significant interactions were found (all p > 0.05).

The attenuation of the response at 8 kHz, equivalent between exposure groups, was most likely related to the difficulties of obtaining reliable DPOAEs at this frequency, rather than attributable to a deficit in OHC function. Responses at this frequency are affected by standing waves in the ear canal (Richmond et al., 2011) and the reflectance magnitude tends to be greatest at 8 kHz (Keefe et al., 1993). These factors, in conjunction, are thought to be responsible for the DPOAE amplitude at 8 kHz often being described as “poor” (Richmond et al., 2011; Gorga et al., 1993, 1997).

### 3.4 ABRs

#### 3.4.1 Effects of session, montage, and group

**Fig. 3:**
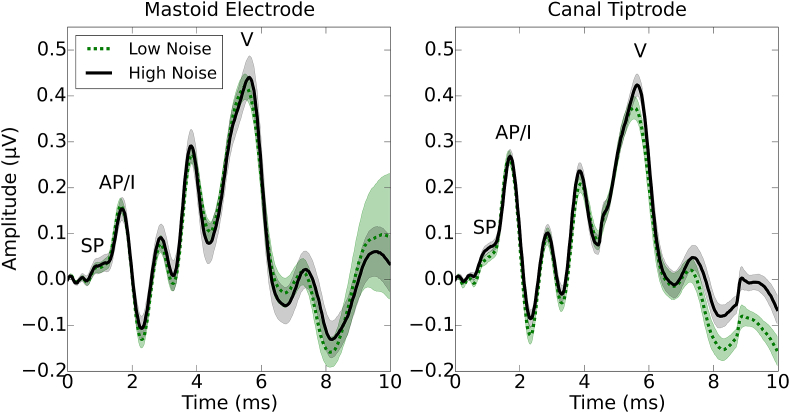
Grand average ABR waveforms in response to a 80 dB nHL click. Waveforms are shown for each group of listeners and for the mastoid electrode and canal tiptrode. 95% confidence intervals are indicated by the shaded areas.

**Fig. 4:**
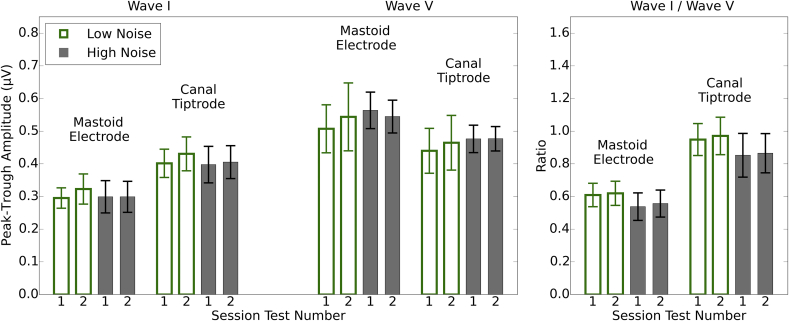
Mean peak-to-trough amplitudes for wave I and wave V, and mean wave I/V ratios. Each test session is plotted individually for the two montages and the two groups. Error bars show 95% confidence intervals.

Fig. 3 shows the grand average ABR waveforms across sessions for the two electrode montages and the two groups of listeners (low- and high-noise exposure). The waveforms appear similar for the two groups. S2 of Supplementary Materials shows the individual waveforms of all 15 listeners in each group, for both electrode montages. Fig. 4 shows the average wave I and wave V amplitudes for the two groups, for each montage and session, together with the I/V amplitude ratios. There is little difference between the groups or sessions. As expected, use of the canal tiptrode montage resulted in larger wave I amplitudes and smaller wave V amplitudes than the mastoid electrode. Equivalent information for wave I and V latency is reported in S3 of Supplementary Materials.

**Fig. 5:**
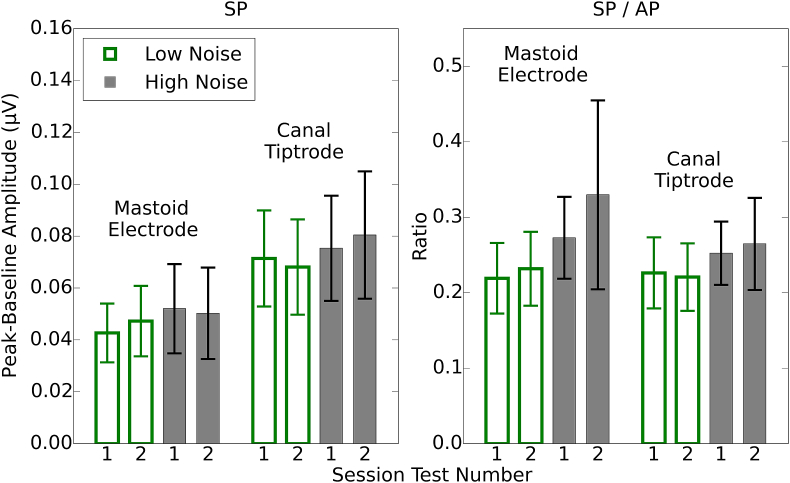
Mean peak-to-baseline amplitudes for the SP and the SP/AP ratio. Each session is plotted individually for the two montages and the two groups. Error bars show 95% confidence intervals.

Fig. 5 shows average SP values for the two groups in each of the sessions, for both montages, and also SP/AP ratios. The SP values are about 50% larger for the canal tiptrode than the mastoid electrode. However, the SP/AP ratios are comparable in size across the two recording montages, with the difference in the montage means ∼0.02.

Mixed design ANOVAs were used to characterize the response amplitudes for each wave of the response, and the ratio measures, separately. Within-subject factors of Test-retest (two levels; T1 and T2) and Montage (two levels; mastoid electrode, canal tiptrode), and a between-subject factor of Group (two levels; low- and high-noise exposure) were included.

For wave I, there was no main effect of Test-retest (F[1,28] = 4.16, p > 0.05) nor Group (F[1,28] = 0.14, p > 0.05). There was a main effect of Montage (F[1,28] = 209.60, p < 0.001) and Bonferroni-corrected post-hoc tests confirm that wave I amplitudes are greater for the canal tiptrode than the mastoid electrode. There are no significant interactions between factors.

For wave V, there was no main effect of Test-retest (F[1,28] = 0.70, p > 0.05) nor Group (F[1,28] = 0.33, p > 0.05). There was a main effect of Montage (F[1,28] = 120.68, p < 0.001) and Bonferroni-corrected post-hoc tests confirmed that wave V amplitudes were greater for the mastoid electrode than the canal tiptrode. There are no significant interactions between factors. The wave I/V ratios showed no significant interactions and no main effect of Test-retest (F[1,28] = 0.09, p > 0.05) nor Group (F[1,28] = 1.58, p > 0.05). As expected, there was a significant main effect of Montage (F[1,28] = 282.52, p < 0.001) with Bonferroni-corrected post-hoc tests indicating that canal tiptrode I/V ratios were significantly greater than the mastoid electrode ratios.

For the SP amplitudes, again there was no main effect of Test-retest (F[1,28] = 0.02, p > 0.05) nor Group (F[1,28] = 0.48, p > 0.05). There was a main effect of Montage (F[1,28] = 55.36, p < 0.001) and Bonferroni-corrected post-hoc tests confirm that SP amplitudes were greater for the canal tiptrode than the mastoid electrode. There were no significant interactions between factors. For the SP/AP ratios there were no significant interactions and no significant main effects of Test-retest (F[1,28] = 0.73, p > 0.05), Montage (F[1,28] = 1.42, p > 0.05), nor Group (F[1,28] = 2.88, p > 0.05).

#### 3.4.2 Reliability

Table 1 shows the CoV for the different wave amplitudes and ratios for the two groups and the two sessions. A lower CoV represents less relative dispersion of the data about the mean. The lowest coefficients were seen for wave V for the high noise exposure group. Overall, CoVs for wave I were similar to those for wave V (all <0.35), and much less than those for the SP. The coefficients for the canal tiptrode were slightly smaller than for the mastoid electrode, by 0.02 and 0.04 for the low- and high-noise exposure groups, respectively. For wave V the high-noise exposure group showed less variability than the low-noise exposure group in both montages. The CoVs for the ratio measures and the SP amplitude were comparable across montages, with the means for each montage differing by no more than 0.1 across all three measures (wave I/V ratio, SP, and SP/AP ratio). The high-noise exposure group showed larger wave I/V ratio variability and greater SP and SP/AP ratio variability.

**Table 1:** CoV values for the two groups of listeners and the two montages for the waves and wave ratios of interest. The value reported is the mean CoV for each of the two sessions calculated independently.

	Mastoid electrode		Canal tiptrode	
	Low noise	High noise	Low noise	High noise
Wave I	0.25	0.32	0.23	0.28
Wave V	0.33	0.19	0.33	0.16
Wave I/V ratio	0.23	0.30	0.22	0.29
SP	0.55	0.67	0.52	0.57
SP/AP ratio	0.42	0.57	0.41	0.39

**Fig. 6:**
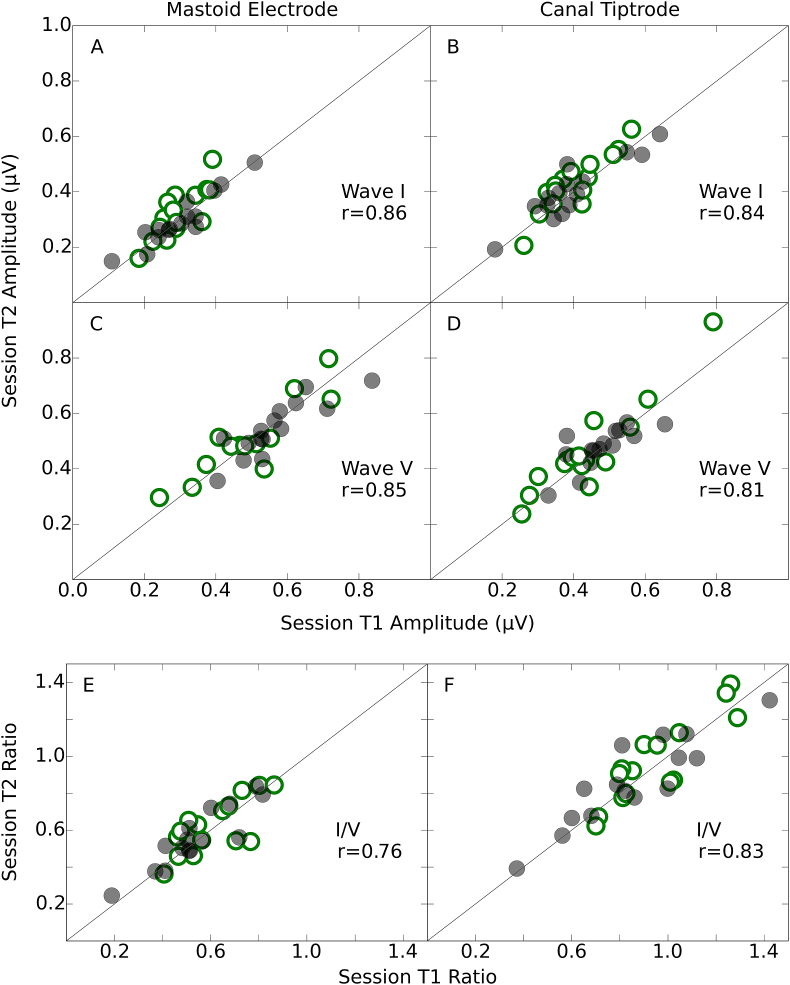
Test-retest reliability of waves I and V, and I/V ratio. Amplitudes and ratios for the second test session (T2) are plotted against those for the first test session (T1). The data for the mastoid electrode and canal tiptrode are plotted in the left- and right-hand column respectively. Spearman correlation coefficients are reported as a summary statistic. Low-noise exposed listeners are shown in open green circles and high-noise exposed listeners in filled grey circles. The diagonal line represents the ideal relation (perfect reproducibility) across both test sessions. (For interpretation of the references to colour in this figure legend, the reader is referred to the Web version of this article.)

Fig. 6 shows wave I and wave V amplitudes for both montages in scatter plots, with session T2 plotted against session T1. The Spearman correlation coefficient was used as a descriptive summary statistic of this relation. The low- and high-noise exposure groups are plotted in different symbols for consistency, but as there were no statistically significant differences between the groups (see section 3.4.1), all correlations and ICCs were computed across all participants. For wave I, the linear correlation between sessions was comparable across the two montages (panels A and B), with a difference of just 0.02. For wave V, the correlation coefficients were 0.04 larger for the mastoid electrode (panel C) than for the canal tiptrode (panel D). The correlation between sessions was as strong for wave I as for wave V. The bottom panel of Fig. 6 shows the wave I/V ratio for session T2 plotted against that of session T1. The correlations for the I/V ratio were larger for the canal tiptrode (panel F) than the mastoid electrode (panel E), and similar to those for the individual waves shown in the upper two panels of Fig. 6.

**Fig. 7:**
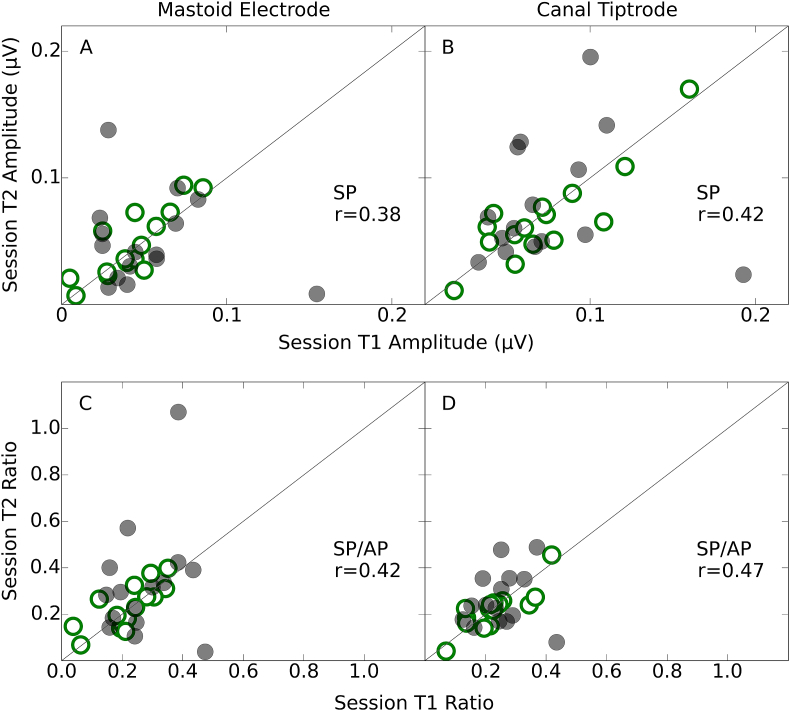
Test-retest reliability of the SP and SP/AP ratio. Amplitudes and ratios for the second test session (T2) are plotted against those for the first test session (T1). The data for the mastoid electrode and canal tiptrode are plotted in the left- and right-hand column respectively. Spearman correlation coefficients are reported as a summary statistic. Low-noise exposed listeners are shown in open green circles and high-noise exposed listeners in filled grey circles. The diagonal lines represent the ideal relation (perfect reproducibility) between test sessions. (For interpretation of the references to colour in this figure legend, the reader is referred to the Web version of this article.)

Fig. 7 shows scatter plots for the SP amplitudes and SP/AP ratios. The correlations between sessions were much weaker for the SP than for the main ABR waves. The correlation coefficients were larger for the SP in the canal tiptrode montage (panel B) than for the mastoid electrode (panel A). The bottom panel of Fig. 7 shows the SP/AP ratios for session T2 plotted against those for session T1. The correlations for the SP/AP ratio were slightly larger in the canal tiptrode montage (panel D), though both recording locations showed much smaller coefficients than the wave I/V ratio.

ICC values are shown in Table 2, together with 95% confidence intervals. The ICCs were largest for waves I, V, and the I/V ratio, and largest for the canal tiptrode montage. These ICC values would generally be described as reflecting excellent repeatability (>0.75; Cicchetti, 1994), both within and between montages. The reliability of wave I across the two test sessions was comparable to that for wave V, with all ICC values greater than 0.80. Wave I amplitudes were larger for the canal tiptrode montage, but it does not appear that this was concordant with a substantial increase in reliability over the mastoid electrode montage. ICC values for wave I and V latency are reported in S4 of Supplementary Materials.

**Table 2:** ICC values for five ABR amplitude measures, both between sessions (for both electrode montages) and between montages. Lower and upper 95% confidence intervals are shown in parentheses.

	Mastoid electrode	Canal tiptrode	Between montage
Wave I	0.85 (0.71/0.92)	0.88 (0.76/0.94)	0.88 (0.80/0.94)
Wave V	0.80 (0.63/0.90)	0.87 (0.75/0.94)	0.90 (0.82/0.95)
Wave I/V ratio	0.84 (0.70/0.92)	0.89 (0.79/0.95)	0.85 (0.74/0.92)
SP	0.18 (−0.18/0.50)	0.40 (0.056/0.66)	0.47 (0.25/0.67)
SP/AP ratio	0.32 (−0.039/0.60)	0.46 (0.13/0.70)	0.31 (0.083/0.54)

The SP and SP/AP ratio measures showed much lower reliability. The SP for the mastoid electrode had poor reliability, and although this was improved by using the SP/AP ratio, it still remained lower than the reliability reported for the other waves. The SP values from the canal tiptrodes were more reliable and these were also improved by using a ratio measure, although, as indicated by the confidence intervals, there was no statistically significant difference between the reliability of the two montages for any of the measured waves or ratios. However, it is clear that any measure utilising the SP was much less reliable than one using waves I and V. The strongest ICC value of the four measures involving the SP (restricted to reliability estimates within a montage) was 0.46. Comparing this ICC value with the weakest ICC from the three measures using waves I and V (0.80) demonstrates that the reliability of measures utilising the SP were significantly poorer than those using waves I and V (z = 2.21; p < 0.05).

## 4 Discussion

### 4.1 Reliability of ABR measures

The primary aim of the current study was to quantify the test-retest reliability of ABR measures, to evaluate whether the ABR is a suitable technique for measuring auditory nerve function in individual human listeners. Although it has been reported that the ABR is stable over long time periods in an individual, much of this evidence relates to wave V. The data presented here indicate that wave I test-retest reliability, and therefore measurement error, is comparable to that of the larger amplitude wave V. Therefore, although wave V is often characterised as robust and reliable, and wave I as small and variable (Mehraei et al., 2016), it is clear that wave I has high within-subject reliability in normal-hearing listeners, at least for the stimulus intensity used here. If the other sources of between-subject variability (for example, head size, tissue resistance) can be controlled, wave I amplitude is sufficiently reliable to accurately characterize individual differences in auditory nerve function.

Neither the SP nor SP/AP ratio were reliable. Even when using the canal tiptrode montage, the best-case ICC was 0.46. In the current study these measures clearly have poor test-retest reliability, but this may be because of the small SP amplitudes evoked by an 80 dB nHL (115.5 peSPL) click. The click used by Liberman et al. (2016) to evoke the SP had a level of 94.5 dB nHL (130 peSPL), and produced much larger SP amplitudes. However, it is not clear that raising presentation levels to enhance the SP is advisable. Even an 80 dB nHL stimulus is intolerably loud for some listeners (Gu et al., 2012). A stimulus presentation level greater than 90 dB nHL (over 120 dB peSPL when presented through ER3A inserts) could risk exceeding recommended daily exposure limits after a few thousand presentations. Moreover, even such exposure limits may be too permissive, since impulse noise is more damaging than continuous-type noise of equivalent energy (Starck et al., 2003). It may also be the case that the SP is inherently unreliable, even if higher stimulus presentation levels are used. Either way, the clinical utility of the SP measure may be limited.

The SP/AP ratio in the current study used an arbitrary baseline to compute the amplitude of both the SP and the AP components, as described by Liberman et al. (2016). It has been reported previously that peak-to-baseline measures of wave I amplitude (the AP) are less reliable than peak-to-trough estimates of amplitude (Stelmack et al., 2003). Therefore, measures such as the SP/AP ratio could benefit from using peak-to-trough estimates of the AP. However, in the current study this made little difference to the reliability of the SP/AP ratio, which suggests that the variability of the SP was the limiting factor.

### 4.2 Effects of electrode montage

One concern when trying to measure small, supra-threshold changes in the auditory nerve function of normal-hearing listeners is that scalp-mounted mastoid electrodes are simply not sensitive enough to reliably detect the subtle changes in evoked responses. The results presented in this study indicate that moving the recording site closer to the generator of wave I (the auditory nerve), by placing a tiptrode in the ear canal, produced only a small increase in reliability for waves I and V, although the benefit was greater for the SP. The amplitude of wave I increased and that of wave V decreased when using a canal tiptrode relative to a mastoid electrode, as seen in other studies (e.g. Bauch and Olsen, 1990). However, reliability of the wave amplitude did not appear to be directly linked to the absolute amplitude of the wave. Wave V was slightly more reliable in the canal tiptrode montage compared to the mastoid electrode montage, despite having lower amplitudes on average. Given that the use of canal tiptrodes increases the financial burden on ABR practitioners and can reduce participant comfort, it is not clear that such equipment is necessary or advisable for the recording of ABR waves I or V.

### 4.3 Relation of ABR measures to noise exposure

The final aim of the study was to investigate supra-threshold changes in the ABR in relation to noise exposure. The results presented here, for a group of young females in which low- and high-noise exposed listeners were well-matched for audiometric thresholds and age, indicate no changes in wave I amplitude as a function of noise exposure. There is no evidence for noise-induced cochlear synaptopathy. This is consistent with other recent studies in our laboratory which have found no association between noise exposure and wave I amplitude in young listeners with normal audiograms (Prendergast et al., 2017; Guest et al., 2017). The range of noise exposures in the present study allowed for good separation between the groups, although compared with Prendergast et al. (2017) there were fewer listeners with very high exposures, and more listeners with very low exposures. It should be noted that an absence of any evidence for cochlear synaptopathy is not the same as evidence for absence of the disorder. It remains unclear how sensitive the ABR is to a loss of low-SR fibers, even in animals (Bourien et al., 2014). Shaheen et al. (2015) suggested that the frequency-following response is a more sensitive identifier of cochlear synaptopathy than the ABR. It may yet prove that in humans, a click-evoked response is too crude a measure with which to elucidate subtle supra-threshold, sub-clinical deficits.

Liberman et al. (2016) also reported no significant difference in wave I amplitude between low- and high-noise exposed groups of listeners, although they did find a large difference between the groups in the SP/AP ratio. Liberman et al. reported mean SP amplitudes of approximately 0.14 and 0.21 μV, and SP/AP ratios of 0.26 and 0.46, for the low- and high-noise exposure groups, respectively. For the canal tiptrode montage in the present study, the SP amplitudes were 0.07 and 0.08 μV, and the SP/AP ratios were 0.22 and 0.26, for the low- and high-noise exposure groups, respectively. Although the present data show a trend in the direction reported by Liberman et al. the effect did not reach significance. The click intensity used in the current study was 14.5 dB lower than that used by Liberman et al. and therefore it may be that substantial differences between noise-exposure groups are only observed for more intense presentation levels than used here. Alternatively, there were substantial high-frequency audiometric differences between the groups in the Liberman et al. study, in contrast to the present study in which the groups were closely matched at high frequencies. Hence the populations tested in the two studies may not be directly comparable. One possibility is that high-frequency audiometric loss is a marker for cochlear synaptopathy. For example, only noise exposures that produce high-frequency threshold elevations may have the capacity to cause a substantial loss of cochlear synapses. Another is that SP/AP ratios may be directly influenced by high-frequency sensitivity, in the absence of synaptopathy. It may also be crucial to consider age more carefully, for example, whether the age at which intense noise exposures are experienced is critical, or whether the effects of noise-induced synaptopathy are more easily observed as an accelerated decline in hearing with advancing age.

## 5 Conclusions

• For young female listeners with normal hearing, ABR wave I and wave V amplitudes, and the I/V amplitude ratio, all show excellent test-retest reliability, with over 80% of the variability in measurement accounted for by between-subject differences in ABR response.
• The SP amplitude and SP/AP ratio show poor levels of reliability for the 80 dB nHL click intensity used here.
• Use of a canal tiptrode may result in slightly improved reliability, although a mastoid electrode is still highly reliable for waves I and V.
• No significant differences were found in any ABR measure between low- and high-noise exposure groups.

## Figures and Tables

**Image 1:**
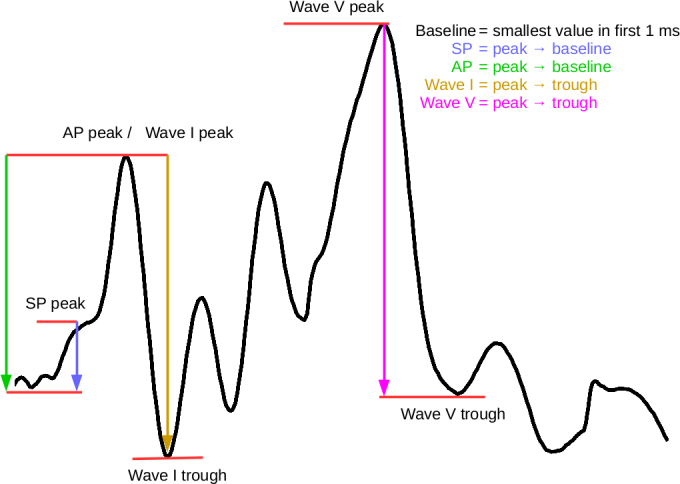


**Image 2:**
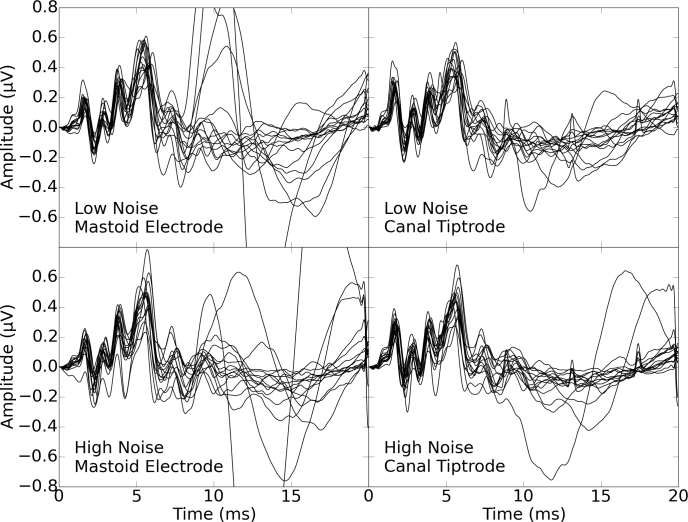


**Image 3:**
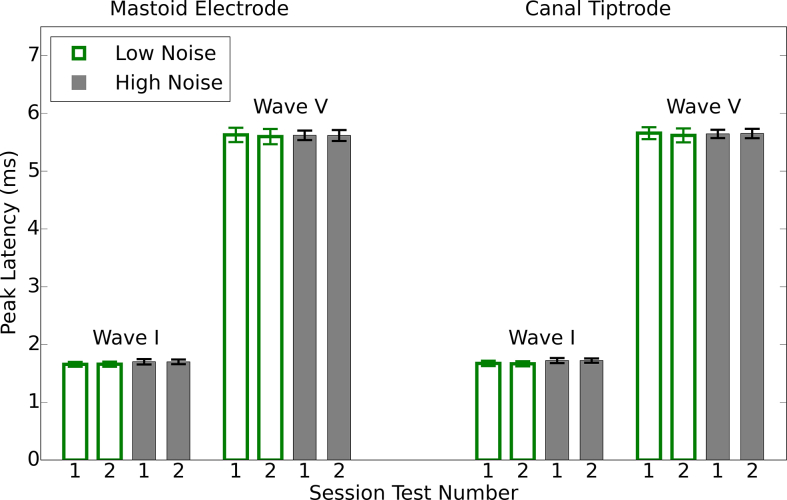


## Appendix A Supplementary data

S4.pdf S4.pdf

SuppMats SuppMats

## References

[bib1] Bauch C.D., Olsen W.O. (1990). Comparison of ABR amplitudes with TIPtrode and mastoid electrodes. Ear Hear..

[bib2] Bidelman G.M., Pousson M., Dugas C., Fehrenbach A. (2017). Test–Retest reliability of dual-recorded brainstem versus cortical auditory-evoked potentials to speech. J. Am. Acad. Audiol..

[bib3] Bourien J., Tang Y., Batrel C., Huet A., Lenoir M., Ladrech S., Desmadryl G., Nouvian R., Puel J.L., Wang J. (2014). Contribution of auditory nerve fibers to compound action potential of the auditory nerve. J. Neurophysiol..

[bib4] Bramhall N.F., Konrad-Martin D., McMillan G.P., Griest S.E. (2017). Auditory brainstem response altered in humans with noise exposure despite normal hair cell function. Ear Hear..

[bib5] British Society of Audiology (2011). Pure-tone Air-conduction and Bone-conduction Threshold Audiometry with and without Masking.

[bib6] Cicchetti D.V. (1994). Guidelines, criteria, and rules of thumb for evaluating normed and standardized assessment instruments in psychology. Psychol. Assess..

[bib7] Edwards R.M., Buchwald J.S., Tanguay P.E., Schwafel J.A. (1982). Sources of variability in auditory brain stem evoked potential measures over time. Electroencephalogr. Clin. Neurophysiol..

[bib8] Fullbright A.N.C., Le Prell C.G., Griffiths S.K., Lobarinas E. (2017). Effects of recreational noise on threshold and suprathreshold measures of auditory function. Semin. Hear..

[bib9] Gorga M.P., Neely S.T., Bergman B., Beauchaine K.L., Kaminski J.R., Peters J., Jesteadt W. (1993). Otoacoustic emissions from nornal-hearing and hearing-impaired subjects: distortion product responses. J. Acoust. Soc. Am..

[bib10] Gorga M.P., Neely S.T., Ohlrich B., Hoover B., Redner J., Peters J. (1997). From laboratory to clinic: a large scale study of distortion product otoacoustic emissions in ears with nornal hearing and ears with hearing loss. Ear Hear..

[bib11] Grinn S.K., Wiseman K.B., Baker J.A., Le Prell C.G. (2017). Hidden hearing loss? No effect of common recreational noise exposure on cochlear nerve response amplitude in humans. Front. Neurosci.

[bib12] Grose J.H., Buss E., Hall J.W. (2017). Loud music exposure and cochlear synaptopathy in young adults: isolated auditory brainstem response effects but no perceptual consequences. Tends in Hearing.

[bib13] Gu J.W., Herrmann B.S., Levine R.A., Melcher J.R. (2012). Brainstem auditory evoked potentials suggest a role for the ventral cochlear nucleus in tinnitus. J. Assoc. Res. Otolaryngol..

[bib14] Guest H., Munro K.J., Prendergast G., Howe S., Plack C.J. (2017). Tinnitus with a normal audiogram: relation to noise exposure but no evidence for cochlear synaptopathy. Hear. Res..

[bib15] Hall J.W. (1992). Handbook of Auditory Evoked Responses.

[bib16] Issa A., Ross H.F. (1995). An improved procedure for assessing ABR latency in young subjects based on a new normative data set. Int. J. Pediatr. Otorhinolaryngol..

[bib17] Keefe D.H., Bulen J.C., Arehart K.H., Burns E.M. (1993). Ear-canal impedance and reflection coefficient in human infants and adults. J. Acoust. Soc. Am..

[bib18] Kim H.-Y. (2013). Statistical notes for clinical researchers: evaluation of measurement error 1: using intraclass correlation coefficients. Restorative Dentistry & Endodontics.

[bib19] Kujawa S.G., Liberman M.C. (2009). Adding insult to injury: cochlear nerve degeneration after “temporary” noise-induced hearing loss. J. Neurosci..

[bib20] Lauter J.L., Loomis R.L. (1986). Individual differences in auditory electric responses: comparisons of between-subject and within-subject variability I. Absolute latencies of brainstem vertex-positive peaks. Scand. Audiol..

[bib21] Lauter J.L., Loomis R.L. (1988). Individual differences in auditory electric responses: comparisons of between-subject and within-subject variability II. Amplitude of brainstem vertex-positive peaks. Scand. Audiol..

[bib22] Liberman M.C., Epstein M.J., Cleveland S.S., Wang H., Maison S.F. (2016). Toward a differential diagnosis of hidden hearing loss in humans. PLoS One.

[bib23] Lutman M.E., Davis A.C., Ferguson M.A. (2008). Epidemiological evidence for the effectiveness of the noise at work regulations. Health and Safety Executive.

[bib24] McGraw K., Wong S.P. (1996). Forming inferences about some intraclass correlation coefficients. Psychol. Meth..

[bib25] Mehraei G., Hickox A.E., Bharadwaj H.M., Goldberg H., Verhulst S., Liberman M.C., Shinn-Cunningham B.G. (2016). Auditory brainstem response latency in noise as a marker of cochlear synaptopathy. J. Neurosci..

[bib26] Munjal S., Panda N., Pathak A. (2016). Long term test-retest reliability of auditory brainstem response (ABR) and middle latency response (MLR). Glob. J. Oto.

[bib27] Prendergast G., Guest H., Munro K.J., Kluk K., Leger A., Hall D.A., Heinz M.G., Plack C.J. (2017). Effects of noise exposure on young adults with normal audiograms I: Electrophysiology. Hear. Res..

[bib28] R Core Team (2015). R: a Language and Environment for Statistical Computing.

[bib29] Richmond S.A., Kopun J.G., Neely S.T., Tan H., Gorga M.P. (2011). Distribution of standing-wave errors in real-ear sound-level measurements. J. Acoust. Soc. Am..

[bib30] Santos M., Marques C., Nobrega Pinto A., Fernandes R., Coutinho M.B., Almeida E Sousa C. (2017). Autism spectrum disorders and the amplitude of auditory brainstem response wave I. Autism Res..

[bib31] Schaette R., McAlpine D. (2011). Tinnitus with a normal audiogram: physiological evidence for hidden hearing loss and computational model. J. Neurosci..

[bib32] Shaheen L.A., Valero M.D., Liberman M.C. (2015). Towards a diagnosis of cochlear neuropathy with envelope following responses. J. Assoc. Res. Otolaryngol..

[bib33] Shrout P.E., Fleiss J.L. (1979). Intraclass correlations: uses in assessing rater reliability. Psychol. Bull..

[bib34] Spankovich C., Griffiths S.K., Lobarinas E., Morgenstein K.E., de la Calle S., Ledon V., Guerico D., Le Prell C.G. (2017). Temporary threshold shift after impulse-noise during video game play: laboratory data. Int. J. Audiol..

[bib35] Starck J., Toppila E., Pyykko I. (2003). Impulse noise and risk criteria. Noise Health.

[bib36] Stelmack R.M., Knott V., Beauchamp C.M. (2003). Intelligence and neural transmission time: a brain stem auditory evoked potentials analysis. Pers. Indiv. Differ..

[bib37] Zaki R., Bulgiba A., Nordin N., Azina Ismail N. (2013). A systematic review of statistical methods used to test for reliability of medical instruments measuring continuous variables. Iranian Journal of Basic Medical Sciences.

