# Supplementary material for: Supra-threshold auditory brainstem response amplitudes in humans: Test-retest reliability, electrode montage and noise exposure
Source: Hear Res. 2018 Jul;364:38–47. doi: 10.1016/j.heares.2018.04.002 (PMC5993871; doi:10.1016/j.heares.2018.04.002)
Supplement: S4.pdf [file mmc1.pdf]

	Mastoid Electrode		Canal Tiptrode	
	Low noise	High noise	Low noise	High noise
Wave I	0.94 (0.85/0.98))	0.90 (0.73/0.96)	0.97 (0.91/0.99)	0.93 (0.81/0.98)
Wave V	0.97 (0.92/0.99)	0.95 (0.86/0.98)	0.92 (0.79/0.97)	0.95 (0.85/0.98)
