# Supplementary material for: Supra-threshold auditory brainstem response amplitudes in humans: Test-retest reliability, electrode montage and noise exposure
Source: Hear Res. 2018 Jul;364:38–47. doi: 10.1016/j.heares.2018.04.002 (PMC5993871; doi:10.1016/j.heares.2018.04.002)
Supplement: SuppMats [file mmc2.doc]

S1. Schematic depicting how each of the wave amplitudes were defined.

S2. Individual ABR waveforms are plotted for all 15 participants in each exposure group. The top row shows the low-noise exposed listeners and the bottom row the high-noise exposed listeners. The left-hand column shows data from the mastoid electrode and the right-hand column data from the canal tiptrode.

S3. Mean peak latencies for wave I and wave V. Each test session is plotted individually for the two montages and the two groups. Error bars show 95% confidence intervals.

S4. ICC values for ABR wave I and V latency for both electrode montages. Lower and upper 95% confidence intervals are shown in parentheses.
